# Morphological stasis in the first myxomycete from the Mesozoic, and the likely role of cryptobiosis

**DOI:** 10.1038/s41598-019-55622-9

**Published:** 2019-12-24

**Authors:** Jouko Rikkinen, David A. Grimaldi, Alexander R. Schmidt

**Affiliations:** 1 Finnish Museum of Natural History, University of Helsinki, P.O. Box 7, 00014 Helsinki, Finland; 2 Organismal and Evolutionary Biology Research Programme, Faculty of Biological and Environmental Sciences, University of Helsinki, P.O. Box 65, 00014 Helsinki, Finland; 3 Division of Invertebrate Zoology, American Museum of Natural History, New York, 10024-5192 USA; 4 Department of Geobiology, University of Göttingen, Goldschmidtstraße 3, 37077 Göttingen, Germany

**Keywords:** Ecology, Evolution

## Abstract

Myxomycetes constitute a group within the Amoebozoa well known for their motile plasmodia and morphologically complex fruiting bodies. One obstacle hindering studies of myxomycete evolution is that their fossils are exceedingly rare, so evolutionary analyses of this supposedly ancient lineage of amoebozoans are restricted to extant taxa. Molecular data have significantly advanced myxomycete systematics, but the evolutionary history of individual lineages and their ecological adaptations remain unknown. Here, we report exquisitely preserved myxomycete sporocarps in amber from Myanmar, ca. 100 million years old, one of the few fossil myxomycetes, and the only definitive Mesozoic one. Six densely-arranged stalked sporocarps were engulfed in tree resin while young, with almost the entire spore mass still inside the sporotheca. All morphological features are indistinguishable from those of the modern, cosmopolitan genus *Stemonitis*, demonstrating that sporocarp morphology has been static since at least the mid-Cretaceous. The ability of myxomycetes to develop into dormant stages, which can last years, may account for the phenotypic stasis between living *Stemonitis* species and this fossil one, similar to the situation found in other organisms that have cryptobiosis. We also interpret *Stemonitis* morphological stasis as evidence of strong environmental selection favouring the maintenance of adaptations that promote wind dispersal.

## Introduction

Myxomycetes or ‘slime molds’ are a monophyletic lineage of eukaryotes that produce intriguing, morphologically complex fruiting bodies. They are phylogenetically an ancient lineage within the Amoebozoa^1–3^, but like most amoeboid microorganisms, direct evidence of their evolutionary history is extremely scarce^4^.

The traditional classification of myxomycetes has been based entirely on morphological characteristics of the fruiting bodies and spores, so molecular methods have recently provided many new insights into phylogenetic relationshipse.g. refs. ^5–10^. In fact, the hierarchical classification of myxomycetes has been revised based on DNA data^5^.

Phylogenetic studies have confirmed that together with dictyostelioids (Dictyosteliomycetes) and three genera of fruiting amoebae (Ceratiomycetes) the myxomycetes (Myxomycetes) represent a monophyletic group within the subgroup Evosea of Amoebozoa^11–13^. These phylogenies have not supported the traditional five-order classification of the myxomycetes that was followed in most previous treatments e.g. refs. ^14–17^. Rather, two main lineages appear to occur phylogenetically within the myxomycetes. One contains the traditional orders Stemonitales and Physarales, which have a columella (extension of the stalk inside the sporotheca) and spores that are darkened due to melanin in the spore walls (Fuscisporidia). The traditional order Echinosteliales tends to have hyaline spores but constitutes a basal group within the dark-spored lineage. The second main clade includes myxomycetes of the traditional orders Licelaes and Trichiales, which have light-colored spores having little or no melanin in the spore walls^7,10^. The peridium, a usually persistent covering that surrounds the spore-containing sporothecae of fruiting bodies, is an apomorphic character for the class Myxomycetes. However, the peridium is evanescent in the genus *Echinostelium*, in most members of the order Stemonitidales (the group to which the amber fossil belongs), and in many Trichiales. The central synapomorphy of the newly circumscribed order Stemonitidales is the fugacious peridium, i.e., the mature sporothecae of these myxomycetes lack peridia^5^.

Here we present fossil evidence of one of the two main lineages of the myxomycetes, in amber approximately 100 million years old from northern Myanmar. The fossil sporocarps are indistinguishable from the extant genus *Stemonitis* and provide unique insight into the longevity of ecological adaptations in myxomycetes. The fossil can be used as a minimum age constraint for this genus after more comprehensive and robust analyses of phylogenetic relationships among the Stemonitidales and related lineages become available.

## Results and Discussion

### Description of the fossil

**Figure 1:**
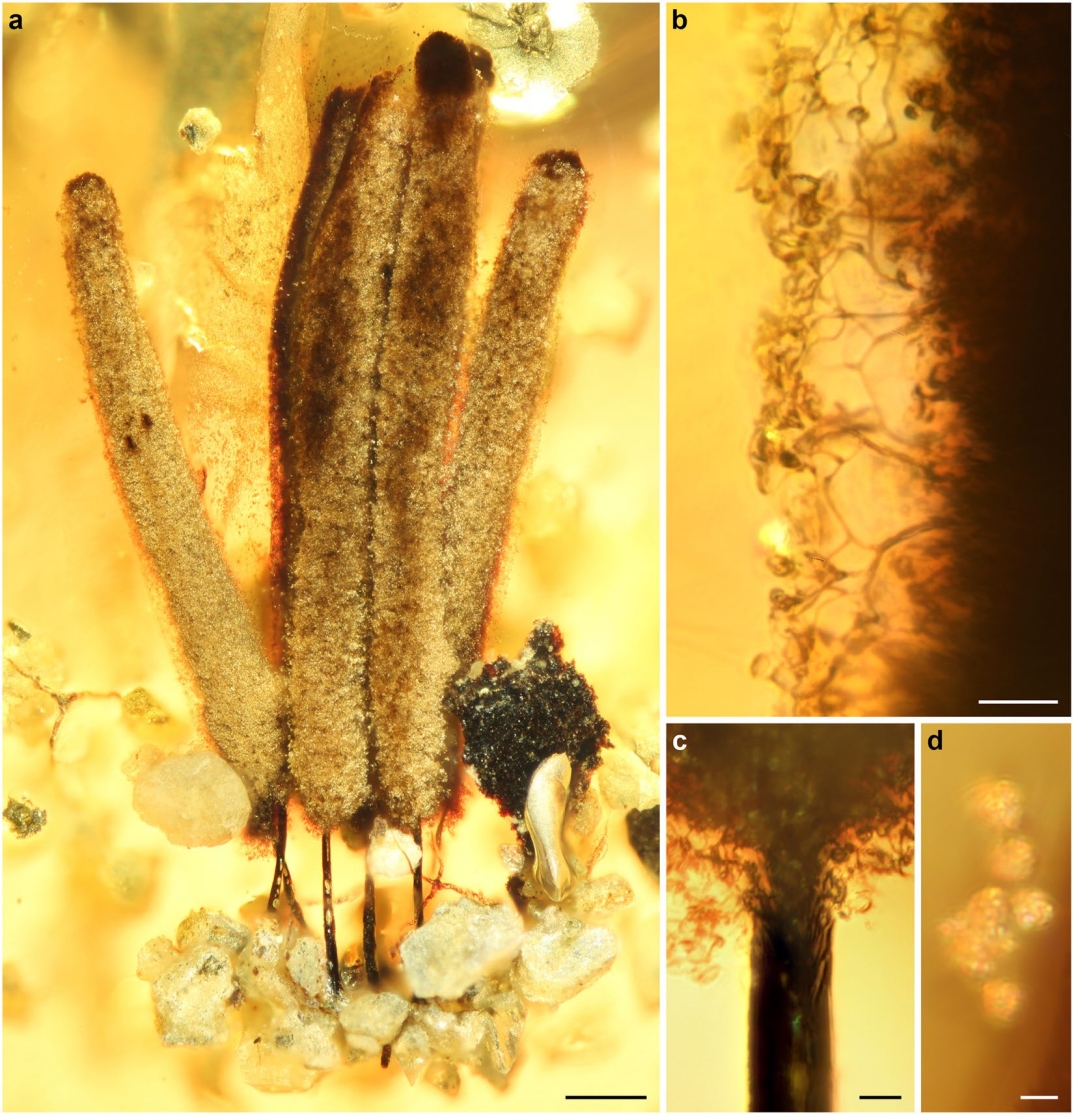
Fossil *Stemonitis* in mid-Cretaceous amber from northern Myanmar. (**a**) General habitus of sporocarps. (**b**) Surface of sporotheca, showing details of capillitium. (**c**) Base of sporotheca, showing stalk continuing as a columella into the sporotheca. (**d**) Detached spores. Scale bars 200 µm in (**a**). 20 µm in (**b**), 10 µm in (**c**), and 5 µm in (**d**).

Entire specimen consists of a densely-arranged group of six stalked sporocarps entrapped in resin while young, with almost entire spore mass still inside sporotheca (Fig. 1). Sporocarps initially in contact, two marginal ones displaced during preservation and leaning outwards. Stalks reddish brown to black, opaque, up to 400 µm in length, without fibrous base; some stalks ruptured indicating a hollow centre. Numerous quartz (sand) grains attached to common base of sporocarps, suggesting that particles attached to thin, sticky hypothallus deposited by plasmodium earlier during fruiting. Sporotheca cylindrical and rounded at both ends, ca. 2 mm long and 150–220 µm wide, blackish brown and becoming translucent after shedding spores; peridium not preserved. Stalk continuing as columella into sporotheca, probably extending past middle, but hidden by dense spore mass and not seen through ruptured apices of two sporocarps. Connection between columella and capillitium only visible at basal ends of spore cases, but capillitium probably connected to columella along its entire length. Capillitium forming dichotomous branches without markedly expanded nodes, giving rise to large meshes before reaching surface. Capillitium threads becoming more slender and paler towards surface where they anastomose to form peripheral surface net with few free ends, consisting of patchwork of small irregular meshes 5 µm across and larger meshes of 20–25 µm across. Spores dark brown en masse, pale brown or translucent in transmitted light, 5–7 µm in diameter, with clearly reticulate surface ornamentation.

### Taxonomy

All morphological characteristics substantiate that the fossil is not just a myxomycete (class Myxomycetes, phylum Eumycetozoa) but indicate placement in the extant genus *Stemonitis* Gled. This is the type genus of the family Stemonitidaceae, order Stemonitidales, superorder Stemonitidia, and subclass Columellomycetidae^5^. The class Myxomycetes is comprised of sporocarpic Eumycetozoa with fruiting bodies containing numerous spores and usually a persistent peridium around the sporotheca (taxa with evanescent peridia lack the covering in mature fruiting bodies)^18^. The subclass Columellomycetidae comprises the dark-spored myxomycetes with a capillitium connected to a true columella. The superorder Stemonitidia have spores appearing usually dark en masse, with order Stemonitales having an epihypothallically developed stalk that usually extends into a true columella, and an evanescent peridium. The family Stemonitidaceae is characterized by a branching and anastomosing capillitium arising from the columella and with capillitial threads usually forming a surface net. Finally, the genus *Stemonitis* unites the myxomycetes that produce cylindrical sporothecae and have a richly branched and anastomosed capillitium that forms a pronounced surface net.

Identification of extant myxomycete species is based almost entirely on morphological characteristics of sporocarps and spores^16,17^. The genus *Stemonitis* currently includes ca. 20 accepted species^19^. Many of them are common worldwide, but also morphologically plastic. Due to considerable morphological variation within extant *Stemonitis* species, a confident infrageneric assignment of the fossil is impossible. However, the relatively small sporocarps with short stalks (ca. 1/5 of sporocarp height) and the relatively small, pale, reticulate spores closely resemble those of the cosmopolitan *Stemonitis virginiensis* Rex^16^.

### Paleoecological setting

Remains of diverse conifers and angiosperms, such as leafy shoots, leaves and flowers, occur as inclusions in Burmese amber^20^. Araucariaceae and Cupressaceae (*Metasequoia*) have been suggested as botanical sources of the fossil resin, respectively^21,22^. Moreover, Burmese amber currently represents the most important source of Cretaceous non-spermatophyte plants. The number of fern taxa in Burmese amber exceeds that of any other amber deposit worldwide^23^ and mosses and liverworts are very diverse in this amber^24,25^, which suggests high humidity and precipitation in the Burmese amber paleoenvironment and agrees with the presence of angiosperm leaves with drip-tips, velvet worms (phylum Onychophora) and other distinctly wet tropical organisms in this amber^20^.

**Figure 2:**
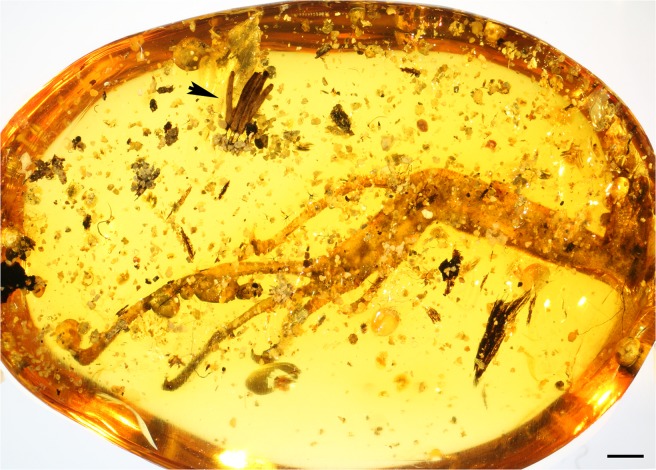
Overview of Burmese amber specimen JZC Bu266 (amber collection of the Division of Invertebrate Zoology, American Museum of Natural History) showing the close proximity of the myxomycete sporocarps (arrowhead) and the hind leg of an agamid lizard. Scale bar 1 mm.

Amber piece JZC Bu266 containing the *Stemonitis* fossil also contains a left posterior (hind) leg of an agamid lizard that retained scale pigmentation, as previously reported (Fig. 2h and 4 h in Daza *et al*.^26^). The leg of the agamid lizard is preserved as an 18 mm-long distal portion with toes and claws, a length almost equal that of the amber piece (Fig. 2). Two toe tips and claws of a second leg are preserved at the edge of the piece, suggesting that the entire lizard was trapped by a resin flow, if not entirely preserved. The shortest distance between the *Stemonitis* sporocarps and one claw of a toe is only 2.2 mm. There are no insects or other obvious syninclusions except for several branched plant trichomes, which are abundant in Burmese amber, as well as tiny fragments of wood or bark remains, hardened resin, and some quartz (sand) grains.

**Figure 3:**
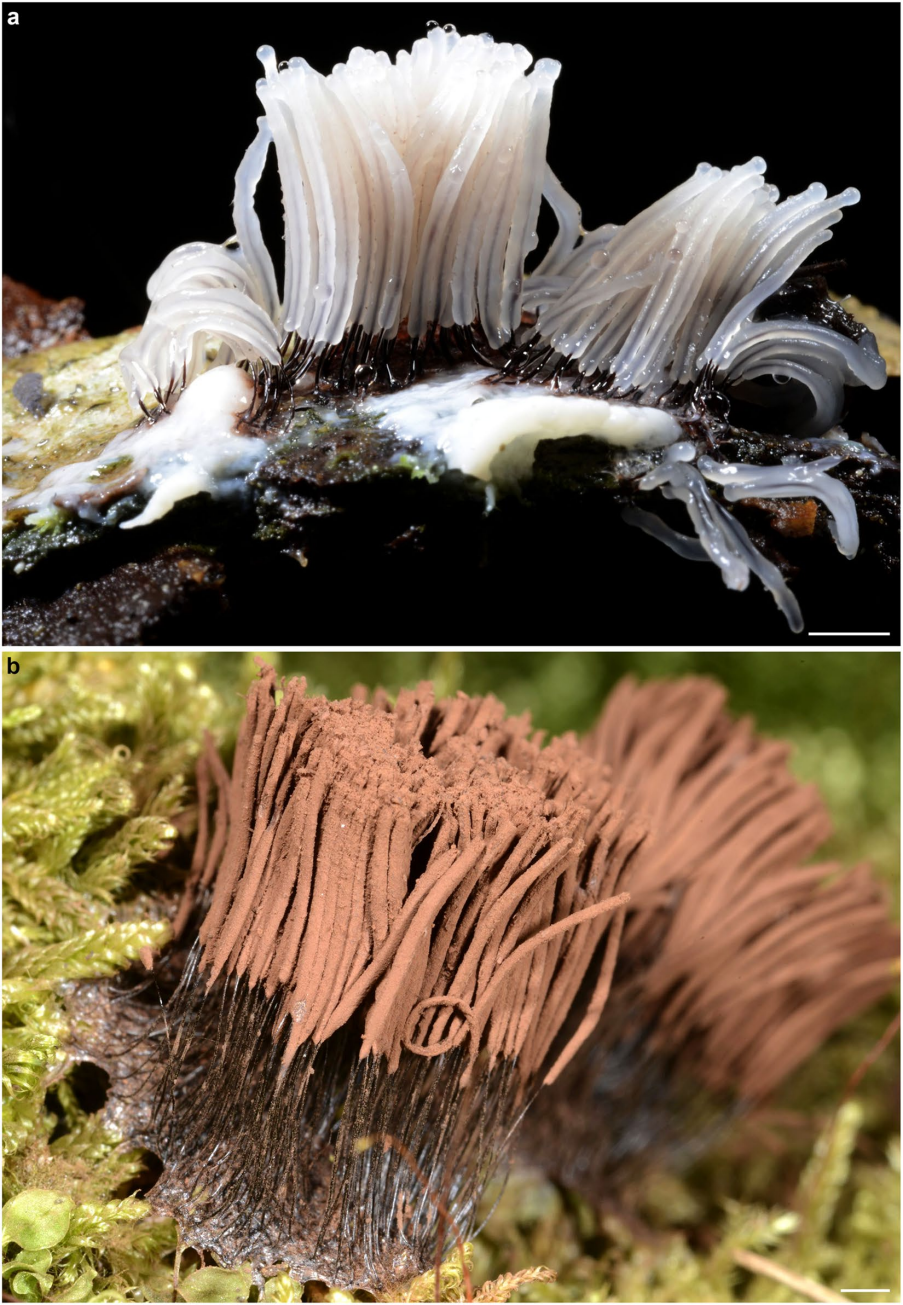
Extant *Stemonitis* species (*Stemonitis axifera*) producing sporocarps on bark and lignicolous bryophytes (Saarijärvi, Finland). (**a**) Young sporocarps. (**b**) Mature sporocarps. Scale bars 2 mm.

Given the small, isolated clump of sand grain substrate and sporocarps, the fossilized *Stemonitis* obviously was not trapped *in situ* but either transported by flowing resin, or perhaps it fell into stationary exudate after being dislodged from the original substrate. Plasmodia of extant *Stemonitis* species are often seen on the bases of tree trunks and on partly decorticated logs, where they also produce their fragile fruiting bodies (Fig. 3). The detachment of a small group of young, dry sporocarps from a common hypothallus must have required some force, and the ruptured apices of two sporocarps indicate mechanical damage. It is feasible that an abrupt detachment may have happened under the foot of a climbing lizard, thrashing from its entrapment in viscous resin.

### Evolutionary implications

The fascinating life cycle of myxomycetes involves two main stages, one consisting of uninucleate amoebae (with or without flagella), and the second a multinucleate plasmodium, which under favorable conditions develops into spore-containing fruiting bodies^27^. The motile amoebae feed on bacteria and divide until they give rise to the plasmodium. The plasmodium, which also moves, feeds by engulfing surface bacteria, fungal spores, yeasts and algae. The amoebae can avoid adverse conditions by transforming into dormant microcysts, and the plasmodium can convert into a hard, dormant sclerotium. Microcysts and sclerotia can withstand drought and cold, and spores may remain viable for years or even decades^28,29^.

Myxomycetes are ubiquitous in most vegetated, terrestrial ecosystems^27,30^. Their diversity tends to be higher in communities with higher diversity and biomass of the vascular plants, which support the microorganismal substrates upon which the amoebae and plasmodia feed. They are most diverse in tropical and temperate forests, and least in boreal forests, arctic or alpine ecosystems. In tropical forests, myxomycete diversity appears to be greater in arboreal microhabitats; in more temperate forests, greater diversity occurs on or close to the forest floor. Moreover, in tropical forests, myxomycetes seem to produce fruiting bodies more readily in relatively dry habitats^27,30,31^.

As myxomycetes spend much of their lives as microscopic spores, unicellular amoebae, or resting cells, and their macroscopic plasmodia and fruiting bodies are small, ephemeral and soft, myxomycetes are improbable candidates for fossilization. Unsurprisingly, fossil myxomycetes are exceedingly rare. Domke^32^ reported several sporocarps of *Stemonitis* and Dörfelt *et al*.^33^ a single sporocarp of *Arcyria* (Trichiales) from Eocene Baltic amber. A further specimen from Baltic amber assigned to *Protophysarum* (Physarales)^34^ likely represents a calicioid fungus of the Mycocaliciales (Ascomycota). A putative plasmodium of a myxomycete reported from Miocene Dominican amber^35^ seems to be a pseudoinclusion, resulting from plant exudate that was immiscible with the terpenoid resin matrix (see Fig. 1I in Thiel *et al*.^36^). The recent report of a structure in Burmese amber, interpreted as a dictyostelioid slime mold^37^ is likewise unconvincing. In addition, fossil spores resembling those of some extant myxomycetes have been found from Oligocene and Pleistocene deposits^38^. Some dark, reticulated spores from the Maastrichtian Deccan Intertrappean beds in India were recently suggested to be slime mold spores^39^, but the fossils are much larger than the spores of extant myxomycetes. Other questionable microfossils identified as solitary and conjugating swarm cells of myxomycetes were previously reported from the same deposit^40^. Thus, the fossil *Stemonitis* reported here is one of the few definitive fossil myxomycetes, and the only unquestionable Mesozoic one. In the future, the fossil will provide a solid minimum age constraint for *Stemonitis*. As of yet, molecular divergence-time estimates are premature because a single fossil is insufficient for calibrating the whole of myxomycetes, as various clades may have evolved at different rates^41^. Furthermore, current molecular phylogenies for myxomycetes are based on only 2–3 genes, and support values for many nodes are low^5^.

The spores of nearly all extant myxomycete species range from 5 to 15 µm in diameter, with most species between 8 and 12 µm. The spores are mainly dispersed by wind, but water and animal dispersal are known for some species^5,27,30,31^. All morphological characters of myxomycete fruiting bodies that affect the dispersal abilities of spores, including the overall structure of the fruiting body, the presence and/or length of stalk, and the degree of capillitium development, must be under strong environmental selection^5,42^. The capillitium is of obvious adaptive significance as it effectively prevents the spore mass against detaching from the stalk and allows spores to disperse separately as they dry out^5^. An interesting adaptive trend in myxomycetes is from solitary to compound fruiting bodies and from stalked forms to sessile forms, typically with corresponding change of spore dissemination mode from wind dispersal, often facilitated by a well-developed capillitium, to dispersal via rainwater and/or insects^43,44^.

The stalks of myxomycete sporocarps elevate spores above the substrate, allowing them to dry out and become airborne. Thus, for species inhabiting substrates that dry out rapidly, the resources required for producing a stalk can be put to other uses^31^. Long stalks are especially beneficial on permanently moist substrates, and some myxomycete species that grow in the humid tropics produce particularly long stalks^27^. During evolution there must have been a substantial selective pressure for maintaining stalked sporocarps, as building a stalk is always likely to take resources from spore production^31^. Terminal velocity, the main indicator for dispersal efficiency, depends critically on spore size, and hydrophobic ornaments of the spore surface influence the adhesion properties of the spore^45,46^. As spore size in myxomycetes is critical and preserved, also other features of the fructification must have been well preserved, including the stalk and columella, and the surface net in *Stemonitis*.

The ability of myxomycetes to withstand adverse conditions through extended dormancy can reduce the amount of phenotypic or adaptive change and may also be a significant factor in explaining the observed stasis in sporocarp morphology. A similar phenomenon has been previously proposed for a fossil tardigrade preserved in New Jersey Cretaceous amber^47^. Many species in the phylum Tardigrada are legendary in their cryptobiotic abilities, being able to survive desiccation for decades, as well as anoxia, extreme temperatures, and gamma radiation^48,49^. Among the most experimentally studied of these tardigrades is the global species *Milnesium tardigradum*, which is very similar to a species in 90 million-year-old amber from New Jersey, USA^47^.

Also several other groups of organisms that have durable dormant stages show remarkable morphological stasis. Cryptobiosis is a life stage in which metabolism is virtually to completely arrested, in response to desiccation, heat, cold and other environmental stresses^50^. Perhaps the best known example of this involves tadpole shrimp of the genus *Triops* (Crustacea: Notostraca), which inhabit vernal pools. As the pools evaporate *Triops* eggs enter into anhydrobiosis (a form of cryptobiosis), which can endure water of sub-boiling temperatures for short periods and complete desiccation for decades^50–52^. Fossils from the Triassic of Germany and France, 205–238 Ma, are very similar to the widespread species *T*. *cancriformis*^53,54^, although Trusheim^54^ classified the German Triassic fossils as the subspecies *T*. *cancriformis minor*. Wagner *et al*.^55^ regarded the German Triassic fossils (237–227 Ma) as a separate species, *Triops minor*, because of slight differences in body proportions among developmental stadia. The cladistic analysis of living and fossil notostracans by Lagebro *et al*.^56^ hypothesized *T*. *minor* as distantly related to living *Triops*, but in that analysis the monophyly of *Triops* was poorly supported (appearing in just 57% of trees), and relationships among species and genera poorly resolved. That *Triops* is phenotypically highly conserved, as is Notostraca in general, is further supported by two other findings: 1. The Devonian *Strudops goldenbergi*, fully recognizable as a crown-group notostracan with a large carapace, 12 pairs of thoracic appendages; and a slender, multisegmented telson with terminal pair of long caudal filaments^56^. 2. Morphologically defined species of the closely related genus *Lepidurus* comprise complexes of cryptic species based on genetic sequences^57^. With little question, *Triops* and *Triops*-like notostracans have existed for at least 230 million years.

Interestingly, cryptobiosis in all of these organisms, including slime molds, appears to result from an ability in the dormant stage to replace cellular water with trehalose^50,58^. Cryptobiosis has probably substantially slowed phenotypic change in tardigrades, notostracans, and slime molds by greatly reducing the number of generations over geological time, but more importantly by being a very effective general adaptation for freeing the organism from environmental stress and the need to develop specialized adaptations^59^.

Finding a *Stemonitis* fossil from the mid-Cretaceous is highly relevant for the ongoing discussion on the ecology and morphological adaptations of myxomycetes, and of slow phenotypic change in organisms with life stages that are cryptobiotic. The presumed paleohabitat of the fossil (a tree trunk in a humid tropical forest), is consistent with the habitat preferences of several extant *Stemonitis* species. All morphological features of the fossil closely mirror those of modern species of the genus, demonstrating that all key features of the sporocarp have remained unchanged for at least 100 million years. This provides overwhelming evidence of strong environmental selection favouring the conserved maintenance of morphological adaptations that specifically promote wind dispersal in this group of myxomycetes (Stemonitales).

## Conclusions

*Stemonitis* in mid-Cretaceous Burmese amber is by far the oldest record of myxomycetes known to date. The morphological characteristics of the fossil are essentially identical to those seen in extant species of the genus, revealing that all key features of the sporocarps have resisted change for at least the past 100 million years. We infer this a result of cryptobiosis, and as evidence of strong environmental selection favoring the maintenance of adaptations that promote wind dispersal in this lineage of myxomycetes.

## Materials and Methods

With approximately 1200 fossil species thus far (half of them described in the past three years)^60^, Burmese amber from Myanmar represents the most important current source of three-dimensionally preserved Mesozoic terrestrial organisms, including fungi, plants, and animals^20^. Delicate cellular structures are often preserved in exceptional fidelity.

The fossil myxomycete is enclosed in a 21 × 15 × 4 mm sized piece of mid-Cretaceous Burmese amber (‘Kachin amber’) which derives from the outcrop near the village of Tanai located on the Ledo Road about 105 km north of Myitkyina in Kachin State, Myanmar. Several lines of evidence point to a mid-Cretaceous age of this so-called Burmese amber^20,22^, and biostratigraphic studies suggest a late Albian age of the amber-bearing sediment^61^, hence the inclusions have an age of approximately 100 million years, with a minimum age of 98 million years (earliest Cenomanian) that is based on recent U-Pb dating of zircons^62^. This age estimate has recently been confirmed by the discovery of an ammonite shell of the genus *Puzosia*, a late Albian–late Cenomanian index fossil, enclosed in a piece of Burmese amber^63^.

Based on the collection date of the amber piece, we exclude that the specimen JZC Bu266 derived from the recently described younger Tilin amber from Myanmar, which is Campanian in age and also has a distinct molecular profile^64^. JZC Bu266 was collected prior to 2013 and first reported in 2016^26^ whereas Tilin amber was first reported in 2018^64^.

Amber specimen JZC Bu266 is part of the amber collection of the Division of Invertebrate Zoology American Museum of Natural History, New York, USA. The prepared amber specimen was mounted on a glass microscopic slide with the upper polished surface oriented horizontally. A drop of water was applied to the upper surface of the amber and covered with a 0.06–0.08 mm thickness glass coverslip (Menzel Inc., Braunschweig) to reduce light scattering from fine surface scratches and to improve optical resolution^65^.

The amber inclusion was examined under a Carl Zeiss AxioScope A1 compound microscope using incident and transmitted light simultaneously. Images were taken with Canon EOS 5D digital camera attached to the microscope. For enhanced illustration of three-dimensional structures, all figures are photomicrographic composites which were digitally stacked from up to 70 individual focal planes, using the software package HeliconFocus Version 6.3.3 Pro^66^.

## Data Availability

The fossil specimen is part of the public collection of the American Museum of Natural History in New York, USA.
